# Tuberculosis Causing a Pectoral Mass Mimicking Malignancy: A Rare Presentation of Tuberculosis

**DOI:** 10.7759/cureus.68377

**Published:** 2024-09-01

**Authors:** Tuhanan Dolmus, Kerem Ensarioglu, Tugce Sahin Ozdemirel, Mehmet Kurus, Seref Ozkara

**Affiliations:** 1 Department of Pulmonary Medicine, Ankara Ataturk Sanatoryum Training and Research Hospital, Ankara, TUR

**Keywords:** muscle tuberculosis, tuberculosis masquerading, soft tissue tuberculosis, chest wall mass, extrapulmonary tuberculosis (eptb)

## Abstract

Tuberculosis is an infectious disease that may involve all systems, with the respiratory system being the most commonly affected. Tuberculosis of the chest wall and its structures is rare, in which malignancy and abscess may be counted among possible differential diagnoses. In this case report, a patient with pectoral muscle tuberculosis will be presented. A 47-year-old female with a history of hypertension and epilepsy was evaluated for a rapidly enlarging painful mass under the left breast, which was later considered a cold abscess. The routine laboratory tests showed elevated C-reactive protein and erythrocyte sedimentation rate. Further radiological imaging with computed tomography confirmed the mass with chest wall involvement. The performed biopsy revealed granulomatous inflammation and subsequent tests confirmed acid-fast bacteria, with later confirmation of *Mycobacterium tuberculosis* in the culture. The patient was treated with an intensive regimen of anti-tuberculosis (TB) drugs consisting of isoniazid, rifampin, pyrazinamide, and ethambutol. After nine months, including a treatment extension due to possible vertebral involvement, the patient showed no symptoms and is under follow-up. Extrapulmonary TB, particularly musculoskeletal TB, comes with many diagnostic challenges due to its nonspecific symptoms. Tissue sampling remains the most important aspect of diagnosis confirmation and treatment planning; hence, TB should always be kept in mind among possible differential diagnoses, especially in endemic regions.

## Introduction

Tuberculosis is an infectious disease that may involve all systems, with the respiratory system being the most commonly affected [1]. It remains the leading cause of death when all causes of mortality are investigated. Regarding non-pulmonary involvement, lymph nodes and bone tuberculosis are considered the primarily involved sites, with lymph node and pleural tuberculosis being the most commonly reported sites in Turkey [1-3]. Tuberculosis of the chest wall and its structures is rare, in which malignancy and abscess may be counted among possible differential diagnoses. In this case report, a patient with pectoral muscle tuberculosis will be presented.

## Case presentation

A female, 47-year-old patient with no smoking history and known hypertension and epilepsy history, was evaluated for a mass under the left breast, which had grown in size within a month, and constitutional symptoms, mainly fatigue, and pain. The breathing pattern was regular during the physical examination, and vitals were within normal range. Under the left breast, a non-fluctuating, solid, and painful lesion with a size of 2x2 cm was palpated. The laboratory results revealed an increased C-reactive protein (CRP) level at 150 mg/dL and an erythrocyte sedimentation rate of 62 mm/hour. The patient had no history of immune dysfunction or other formerly known infectious disease. The initial chest X-ray performed during the evaluation was deemed normal, with no pathological findings on lung parenchyma and hilar structures.

**Figure 1:**
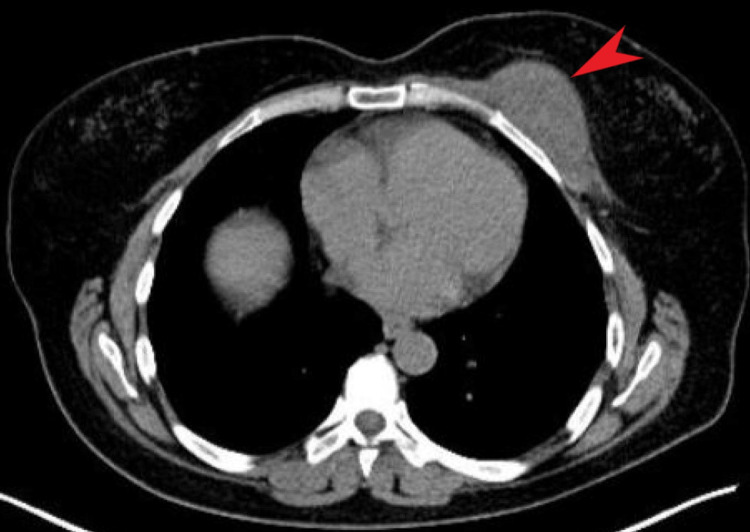
Positron Emission Tomography Chest Imaging In this section of PET-CT, a mass in the left chest can be seen (red arrow), with suspicion for minimal invasion of the underlying bone structures and intrusion into the breast tissue.

For further investigation, computed tomography of the chest and lung was requested to evaluate the mass's nature and whether it was involved in the chest wall. A mass was observed in the lower right quadrant of the left breast, with an estimated size of 27x21 mm, and chest wall involvement was confirmed. An 18-FDG positron emission tomography (PET-CT) was planned for malignancy exclusion, in which an elevated level of SUVmax at 11.9 was reported (Figure 1).

**Figure 2:**
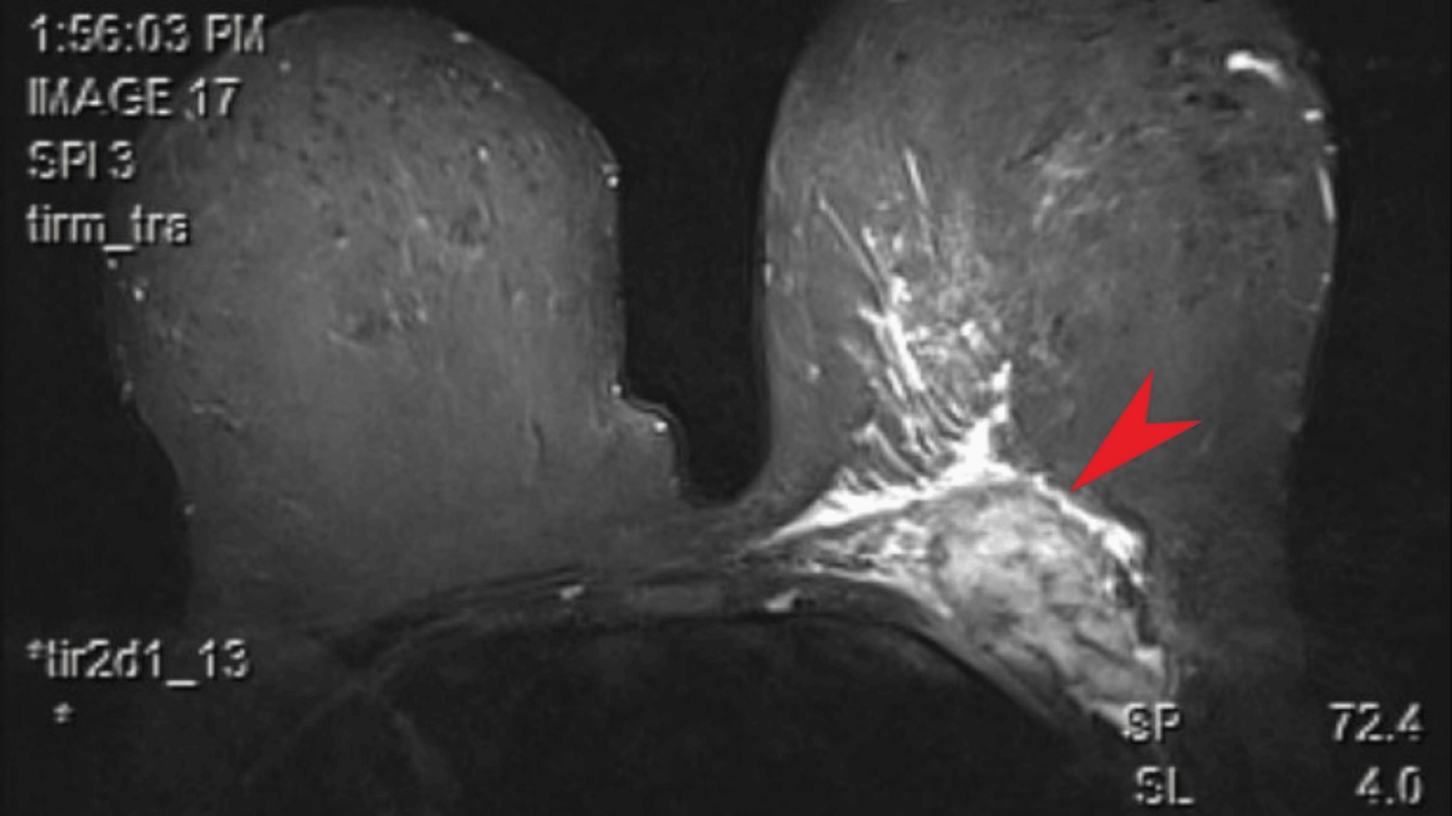
Breast Magnetic Resonance Imaging In the requested follow-up imaging for the tissue involvement of the lesion, involvement of the soft tissue and encapsulated nature of the mass (red arrow), with protrusions to the breast itself, can be seen.

A surgical resection plan was then considered, with the goal of removing the mass and reconstructing the chest wall. A chest wall magnetic resonance imaging (MRI) was requested before the surgery to estimate the resection. Compared to the PET-CT, the MRI results showed the mass to be more clearly defined, with a total size of 4x6x6 cm and edema present peripherally. The underlying involvement of the chest wall in addition to the breast tissue, could be more clearly observed (Figure 2).

In the described lesion, a true-cut needle biopsy was performed, and the sampling was repeated after an initial diagnosis of granulomatous inflammation. As for the definite report, both sampling results were accepted as serious granulomatous inflammation. The additional aspirated liquid confirmed the presence of acid-fast resistant bacteria in favor of mycobacterium in the smear of the aspirate. In between two tests, tumor markers were also requested with no positive results. The diagnosis of pectoral muscle tuberculosis was then confirmed, with the exclusion of lung tuberculosis by sputum sampling. An intensive treatment regimen of rifampicin, ethambutol, pyrazinamide, and isoniazid was then initiated. During the treatment period, the diagnosis was reconfirmed with the culture positivity for tuberculosis.

**Figure 3:**
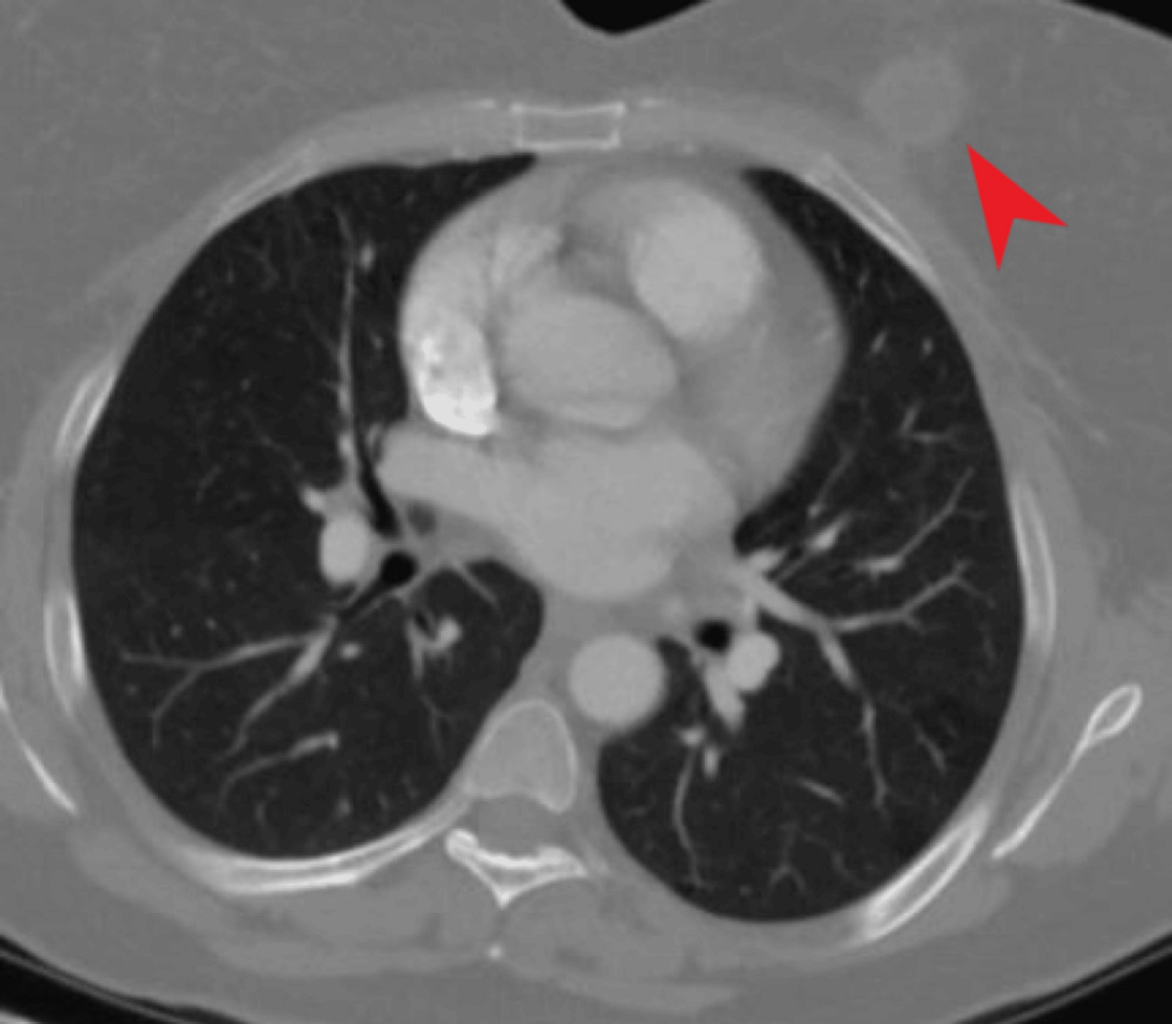
Follow-Up Chest Tomography After treatment, the mass of the left breast had been significantly reduced in size (red arrow).

In the first weekly evaluation, the patient had elevated levels of liver enzymes, with AST and ALT at four times the normal range. The treatment was continued for another week, as the patient was not symptomatic in terms of liver injury. However, a revision was later required as the liver enzyme elevation was persistent and above five times the normal range. After a regimen change to moxifloxacin, cycloserine, streptomycin, ethambutol, and supplementary B6 vitamin, the liver enzymes were found at a normal level after two weeks. The original regimen was re-initiated at the same dosage, with no symptoms and laboratory abnormalities reported afterward. The intensive regimen lasted for two months, with the maintenance treatment of rifampin and isoniazid given for four months. At the end of six months, the patient was reevaluated. The former imaging reports of the CT scans were investigated, and due to possible vertebral involvement at the T10 level, the total treatment duration increased to nine months. The treatment ceased at this point, and afterward, the patient did not report any symptoms and is currently on routine follow-up (Figure 3).

## Discussion

The definition of extrapulmonary TB (EPTB) consists of tuberculosis presence in organs other than the lungs [2,3]. Common organ involvement includes cranial, lymphadenitis, genitourinary, and miliary forms, with pleural involvement being Turkey's most commonly observed one. Coinfection of lung and extrapulmonary systems, such as lung and miliary infection, may also be present, with multiorgan involvement reported at 5.8% in Turkey. Tissue sampling is often required in many cases, with fine-needle aspiration being utilized in many forms, such as endobronchial ultrasonography for mediastinal lymph nodes or pleural sampling. While clinical presentation takes priority in diagnosis, confirmation of the infection by sampling is essential in EPTB to exclude other granulomatous diseases and possible malignancies. Tissue sampling also allows molecular testing and culturing for possible drug resistance [2].

Musculoskeletal TB (MSTB) is a common form of EPTB and may remain undiagnosed for a long period due to non-specific symptoms [4]. Bone involvement is the more commonly encountered form compared to muscular tissue TB, in which nearly all bone, joint, or bursa could be infected. The vertebral bones are the most prevalent location of bone TB (also defined as Pott’s disease), and similar to other bone involvement, a treatment duration of nine months is required for standard antituberculosis treatment [4]. An isolated presence of muscle tuberculosis is quite rare, with coinfection of bone tissues often present [3]. For imaging modalities, conventional radiography and computed tomography are the mainstay approaches [5]. However, magnetic resonance imaging (MRI) is generally accepted as the modality of choice for soft tissue evaluation. As seen in our case, however, CT scans remain adequate in terms of bone involvement. A combination of these methods often allows a better view of the extent of the disease, as seen in this case, in which a bone involvement was present despite the soft tissue being affected predominantly. This could have been attributed to a paradoxical reaction of the bone lesion, as the lesions had become more evident after the initial treatment. The rarity of isolated muscle involvement as the initial presentation of TB often causes a delay in diagnosis, as seen in this case report, and the disease is usually diagnosed after another tissue or organ system is affected or after an intensive differential diagnosis effort is performed.

## Conclusions

TB infection remains one of the most unique clinical presentations that may be observed in the human race. Its ability to mimic many other significant and insignificant pathologies, along with varying latent periods, creates confusion and delay of diagnosis in many cases. While some entities are well known, such as TB of the breast with granulomatosis mastitis, rarer presentations are still possible, like the one in this case report. Here, TB mimicked not only a possible pectoral mass but also its own possible presentation as breast involvement, which further complicated the diagnostic process. Hence, TB must always remain an entity in mind regarding differential diagnosis of mass, especially in endemic regions.
